# Abdominal obesity and digestive system cancer: a systematic review and meta-analysis of prospective studies

**DOI:** 10.1186/s12889-023-17275-2

**Published:** 2023-11-27

**Authors:** Xue Li, Yajun Lian, Weiwei Ping, Kunbo Wang, Lingyan Jiang, Shaoxia Li

**Affiliations:** 1 School of Public Health, Shanxi Medical University, Taiyuan, China; 2 Heping Hospital Affiliated to Changzhi Medical College, Changzhi, China; 3 Department of Public Health and Preventive Medicine, Changzhi Medical College, 161 Jiefang East Street, Changzhi, 046000 Shanxi China; 4 Xiangya School of Public Health, Central South University, Changsha City, China

**Keywords:** Abdominal obesity, Waist circumference, Waist-to-hip ratio, Digestive system cancer

## Abstract

**Background:**

The diagnostic criteria for abdominal obesity are usually waist circumference or waist-to-hip ratio. The magnitude of the risks for cancers of the digestive system and abdominal obesity is unknown. To assess whether abdominal obesity increases the risk of digestive cancer, we conducted a systematic review and meta-analysis of prospective cohort studies in a database.

**Methods:**

PubMed, Embase, and Web of Science databases were searched from their inception to December 2022. The 9-star Newcastle Ottawa Scale was used to assess  study quality. Pooled relative risks and 95% confidence intervals were calculated using fixed or random effect models respectively. The stability of the results was explored by one-by-one exclusion. Subgroup analysis was conducted to explore sources of heterogeneity. Publication bias was evaluated by Begg’s and Egger’s tests.

**Results:**

A total of 43 cohort studies were included. There were 42 and 31 studies in the meta-analysis of waist circumference and waist-to-hip ratio on digestive system cancer, respectively. The results of the meta-analysis revealed that the greater waist circumference and waist-to-hip ratio were correlated with increased incidence of digestive system cancers: waist circumference: RR 1.48, 95% CI 1.38-1.59, *p *< 0.001; waist-to-hip ratio: RR 1.33, 95% CI 1.28-1.38, *p = *0.001. Subgroup analysis by cancer type showed that higher WC and WHR would increase the prevalence of LC, PC, GC, EC, and CRC. The sensitivity analysis was conducted by a one-by-one elimination method, and the results of the meta-analysis remained stable. It is proved that the results were robust by the trim-and-fill method.

**Conclusions:**

There was evidence to suggest that abdominal obesity increased the incidence of digestive cancer, it is necessary to take appropriate measures to reduce abdominal obesity. Waist circumference and waist-to-hip ratio may be better predictors of digestive system cancers. However, the association between waist circumference and digestive system cancer was greater, so more attention should be paid to measuring abdominal obesity with waist circumference.

**Supplementary Information:**

The online version contains supplementary material available at 10.1186/s12889-023-17275-2.

## Introduction

Obesity can lead to many chronic diseases such as diabetes, atherosclerosis, tumors, and hypertension. In recent years, obesity has become a growing global public health problem. Epidemiological researches suggest that obesity increases the risk of a variety of tumors, most of which are digestive system tumors [1]. Digestive system cancer (DSC), which mainly includes cancer of the stomach, esophagus, liver, pancreas, and colorectum, has been the main cause of death in the world [2]. In 2022, it is predicted that there will be 1,918,030 cancer cases in the United States, including 343,040 cancers related to the digestive system [3]. According to cancer statistics in 2020, colorectal cancer (CRC), gastric cancer (GC), liver cancer (LC), and esophageal cancer (EC) are among the top 10 major cancers in the world. There were 604,000 new cases of EC, more than 1.0 million cases of GC, 906,000 new cases of LC, and 1.9 million new cases of CRC [4]. Therefore, the risk factors of DSC have received more and more attention.

A lot of observational studies indicate that the abdominal obesity may be more likely to predict the risk of chronic diseases than body mass index (BMI) [5–8]. Moreover, a large number of studies have shown that abdominal obesity is closely related to DSC. A case-control study showed that abdominal obesity increased the risk of EC and GC, independent of BMI [9]. Studies by Maina and colleagues using Mendelian randomization analyses have shown that abdominal obesity as measured by waist-to-hip ratio (WHR) is a more important etiologic risk factor for PC than overall obesity [10]. In a 7-year prospective cohort study of nearly 22.9 million Korean adults, abdominal obesity as measured by Waist circumference (WC) increased the incidence of cancer in various parts of the digestive tract [11]. In the comprehensive evaluation of obesity-related digestive diseases, the Nam found that abdominal visceral obesity increased a series of DSC, such as PC, LC, CRC, and EC [12]. In a study of 33,230 men followed for 14.4 years by Matthews and colleagues, abdominal obesity was strongly associated with an increased risk of DSC [13]. Whether abdominal obesity is associated with overall DSC. Studies on the mechanism of adipose tissue and DSC have shown that adipose tissue is a highly heterogeneous endocrine tissue that can promote metabolic and inflammatory responses. DSC grew anatomically near the adipose tissue. When adipocytes interact with cancer cells, they may dedifferentiate into preadipocytes or cancer-associated adipocytes. These differentiated adipocytes secrete adipokines that stimulate tumor cell adhesion, migration, and invasion [14].

Previous meta-analysis found that abdominal obesity increased the incidence of PC, LC, GC, EC and CRC [15–18]. However, in a meta-analysis of abdominal obesity and PC [15], the incidence of PC was not studied for WC and WHR in the highest category compared with WC and WHR in the lowest category. Meta-analysis about LC [16] only investigated WC, and the combined results of retrospective and prospective studies were used in the same analysis. Retrospective studies may have recall bias, so the results are not as stable as prospective cohort studies. Meta-analysis of GC, EC, and CRC did not include the latest prospective cohort studies [17, 18].

Although abdominal obesity has been consistently associated with an increased risk of DSC, individual studies often do not have enough persuasive power. Moreover, no comprehensive meta-analysis has summarized the magnitude of the association between abdominal obesity and DSC. WC and WHR are the indicators used to measure abdominal obesity [19]. Therefore, the objective of our systematic review and meta-analysis was to further comprehensively understand and quantitatively assess the association between DSC and abdominal obesity defined by WC and WHR. The clinical significance of our meta-analysis has two main aspects. In the first aspect, comprehensively explore the correlation between abdominal obesity and the incidence of DSC, and positively affect the intervention measures to provide reference for reducing the incidence of DSC. Secondly, finding out which of WC or WHR is more relevant to DSC in order to better measure abdominal obesity in clinical practice for the prevention of DSC.

## Methods

### Search strategy

Our systematic review and meta-analysis was conducted about Preferred Reporting Items for Systematic Reviews and Meta-analyses (PRISMA) guidelines [20]. Two independent researchers (XL and YL) searched the Web of Science, PubMed, and Embase databases from their inception to December 2022. In case of any disagreement, it will be settled by discussing or negotiating with a third person (KW). Retrieved the relevant literature in the database and used the following search terms: (abdominal obesity OR central obesity OR obese OR abdominal adiposity OR obesity OR abdominal fat OR waist-to-hip ratio OR waist-hip ratio OR waist circumference OR abdominal adiposity measures OR adiposity measures) AND (digestive system cancer OR stomach neoplasm OR gastric neoplasm OR cancer of stomach OR gastric cancer OR stomach cancer OR cancer of the stomach OR esophageal neoplasm OR esophagus neoplasm OR cancer of esophagus OR esophagus cancer OR esophageal cancer OR liver neoplasm OR hepatic neoplasm OR cancer of liver OR hepatocellular cancer OR hepatic cancer OR liver cancer OR cancer of the liver OR pancreatic neoplasm OR pancreas neoplasm OR cancer of pancreas OR pancreas cancer OR pancreatic cancer OR colorectal neoplasm OR colorectal tumor OR colorectal cancer OR colorectal carcinoma OR colonic neoplasm OR colon neoplasm OR cancer of colon OR colon cancer OR cancer of the colon OR colonic cancer OR colon adenocarcinoma OR rectal neoplasm OR rectum neoplasm OR rectal tumor OR cancer of rectum OR rectal cancer OR rectum cancer OR cancer of the rectum) AND (prospective cohort OR follow up). Search strategies were not limited by language, publication time, or article type. We also searched for relevant comments or references to find other studies that meet the requirements.

### Study selection

The inclusion of the research contains all the requirements showed below: (a) prospective cohort study; (b) the diagnostic criteria for abdominal obesity were WC and/or WHR; (c) the results were measured in the incidence of DSC; (d) relative risk (RR) or hazard ratio (HR) with 95% confidence interval (CI) were available.

Any of the following criteria shall be excluded: (a) the outcomes were recurrence and mortality rates of digestive cancers; (b) the study could not provide complete data; (c) WC and/or WHR did not compare high and low categories.

### Data extraction and quality assessment

Two researchers independently screened the literature (XL and YL). In case of any disagreement, it will be settled by discussing or negotiating with a third person (KW). From the literature searched in the database, duplicate literature was first removed. Second, the title, abstract and literature type of the articles were scanned, and other studies unrelated to the topic were excluded. Finally, the remaining literature was screened by carefully reading the full text, and literature studies were identified for inclusion and analysis.

Two researchers (XL and YL) conducted data extracted, quality assessed, and cross-checked. In case of any disagreement, it will be settled by discussing or negotiating with a third person (KW). Standard data extraction tables were used to extract data from each study, the data included the last name of the first author, year of publication, country, duration of follow-up, gender of the group, age of the group, the total number of people included, the number of cases occurred, measurement of abdominal obesity, risk effect values and 95% CI after adjustment for confounding factors. Study quality was assessed using the 9-star Newcastle Ottawa Scale (NOS) [21]. According to quality criteria, each study was judged on the selection of the study group (4 stars), comparability of the groups (2 stars), and quality of the outcome (3 stars) for a total score of 9 stars. Studies with a score of 7 or more were considered adequately conducted.

### Data analysis

In this study, RR was used as the effect analysis statistic for dichotomous variables, and 95% CI was provided for each effect size. And HR was directly considered as RR [22]. We used of Q test and I² to evaluate the heterogeneity of studies. For the Q statistic, *p* < 0.10 was considered statistically significant. When *I*² =0, it indicates that no heterogeneity is observed, and the greater the *I*² statistic, the greater the heterogeneity. The low, medium and high degree of heterogeneity were represented by *I*² statistics of 25%, 50% and 75%, respectively. If *I*² > 50%, there is obvious heterogeneity [23]. When *I*² < 50%, meta-analysis was carried out using a fixed effect model; otherwise, the random effect model was used [24]. If30tistical heterogeneity exists among studies, subgroup analysis can be better to analyze the origin of heterogeneity [17]. Sensitivity analysis was carried out by the one-by-one elimination method. The publication bias of the studies was estimated by Egger’s test and Begg’s test [25]. If there were publication bias between studies, the trim-and-fill method was used to further evaluate [26]. In order to better reduce the selection bias and bias in the process of data extraction, two researchers were selected to retrieve and extract data at the same time, and a third person was needed to control the bias in case of different opinions. STATA software, version 15.0. was used for all statistical analyses in this study. For statistical significance, the two-tailed *p*-value was less than 0.05.

## Results

### Study selection

**Fig. 1:**
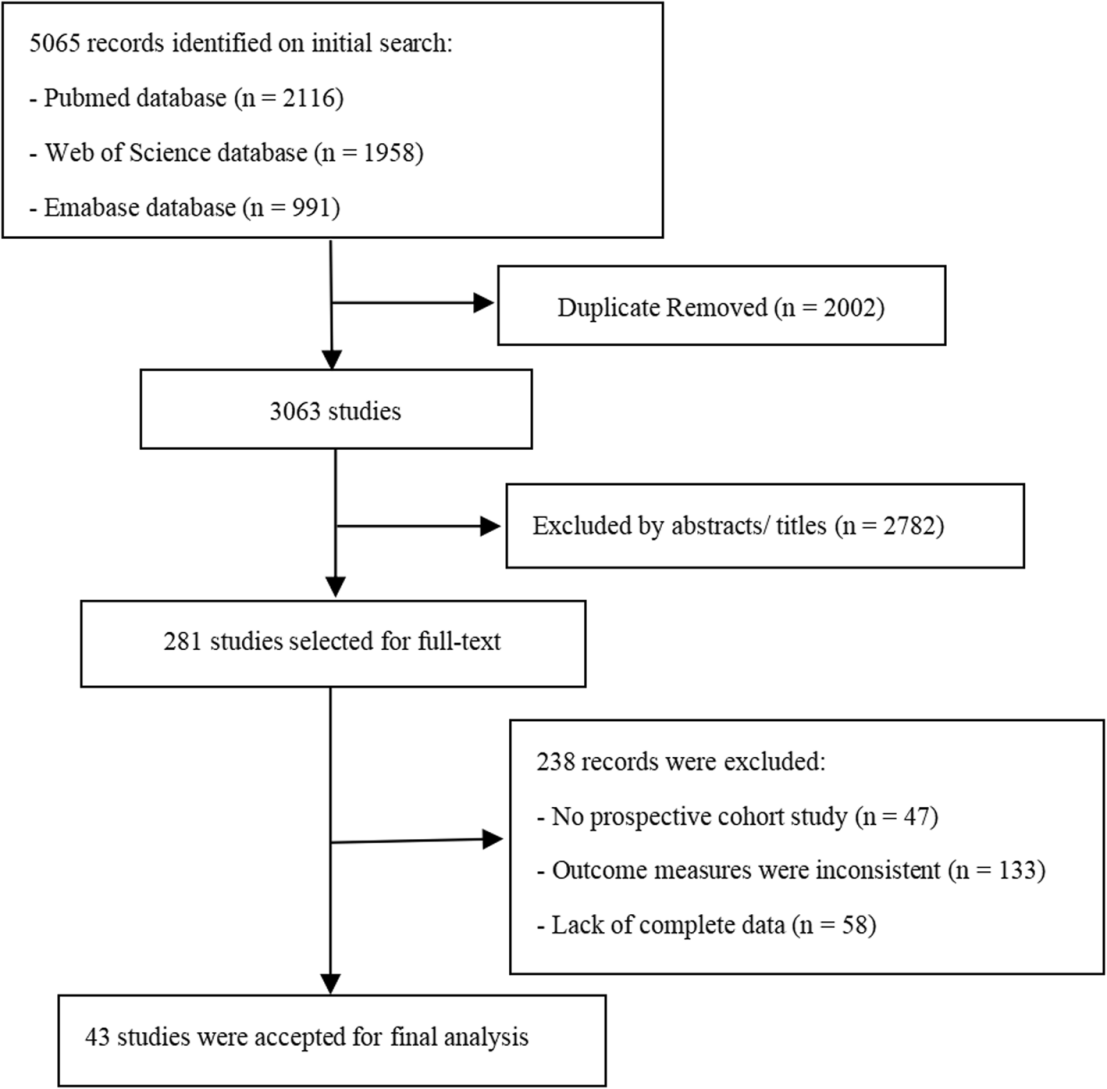
The flow chart of study selection

Five thousand sixty-five​​ records were searched from the databases, 2002 duplicate records were deleted, and 2782 records that were inconsistent with the theme were excluded through reading the abstract and title. 281 records were included in the preliminary screening. Records from 47 non-prospective cohort studies were rejected after reading the full text, 133 records with inconsistent outcome indicators were excluded, and 58 records lacked complete data. Read the full text carefully as requested, a total of 43 qualified studies [27–69] were finally included. There were 42 and 31 studies in the meta-analysis of WC and WHR on DSC respectively. The flow chart of study selection is shown in Fig. 1.

### Study characteristics

All the studies were prospective cohort studies published between 1997 and 2022. Most of the research came from the United States, Europe, and China. Results of all studies were regulated for a series of hidden risk factors, including age, gender, education level, alcohol consumption, smoking, and so on. A total of 25,745,153 people were incorporated into the study on the association between WC and DSC, including 103,590 patients with DSC. A total of 7,805,792 people were incorporated into the study on the association between WHR and DSC, including 29,435 patients with DSC. Table 1 shows the general features of the contained studies. Literature quality was assessed by 9-star NOS, all studies reached a score of ≥ 7 (Table 2).

**Table 1:** Baseline characteristics of included studies regarding WC and risk of DSC

Study (year)	Cancer type	Country	Study population (age)	Duration Of Follow up(year)	Cases/cohort size	Measure of adiposity	Categories, Highest vs. Lowest (Measurement Unit)	Adjusted R (95%CI)	Adjustments
Stolzenberg (2008) [27]	PC	USA	Men and women (50-71 years)	5	654/495035	WC	Q5 vs. Q1	Men:1.07 (0.69, 1.64) Women:2.52 (1.33, 4.77)	Age, smoking status, race , energy (quintiles), energy-adjusted total fat (quintiles), self-reported diabetes , sex.
						WHR	Q4 vs. Q1	Men:1.34 (0.86,2.08) Women:1.19 (0.66,2.15)	
Berrington (2006) [28]	PC	European countries	Men and women (19-84years)	9	324/438405	WC	Q4 vs. Q1	1.14 (0.79-1.63)	smoking status, diabetes and by sex-specific height quartile.
						WHR	Q4 vs. Q1	1.33 (0.93-1.92)	
Luo (2008) [29]	PC	USA	women (19-84years)	7.7	251/138503	WC	Q5 vs. Q1	1.1 (0.7–1.6)	age, different treatment assignments in clinical trials, smoking status and diabetes history at baseline.
						WHR	Q5 vs. Q1	1.7 (1.1–2.6)	
Larsson (2005) [30]	PC	Swedish	Men and women	7	136/83053	WC	Q4 vs. Q1	Men and women: 1.72 (0.93-3.20) Men:2.00 (0.85-4.66) Women:1.46 (0.58-3.66)	age, education, physical activity, cigarette smoking, sex.
Arthur (2018) [31]	PC	USA	Women	17.9	1045/156218	WC	≥95cm vs.<76cm	1.38 (1.14-1.66)	age, smoking status, pack-years of smoking, alcohol intake, metabolic equivalent task hrs/week, educational level, race, and allocation to the OS or treatment/placebo/control arm of clinical trials unless included as main exposure. previous history of diabetes.
						WHR	≥0.86 vs.<0.76	1.40(1.17-1.68)	
Andersson (2016) [32]	PC	European	Men and women (44-73years)	7	163/28098	WC	Q3 vs. Q1	Men and women: 1.58 (0.97-2.57) Men:0.90 (0.47-1.75) Women:1.35 (0.81-2.26)	sex, age, smoking, alcohol consumption, and diabetes.
						WHR	Q3 vs. Q1	Men and women: 2.36 (1.28-4.35) Men:1.35 (0.71-2.57) Women:1.35 (0.81-2.26)	
Hwang (2021) [34]	LC	Korea	Men and women(≥20year)	7.3	26979/9671941	WC	Men:≥105cm vs. 85-<90cm Women:≥100cm vs.80-<85cm	Men and women: 1.69 (1.54-1.85) Men:1.76 (1.57-1.97) Women:1.56 (1.32-1.84)	age, sex, alcohol intake, smoking, physical activity, income status, diabetes, hypertension, dyslipidemia,liver cirrhosis and viral hepatitis.
Campbell (2016) [35]	LC	USA	Men and women	ND	2162/1570000	WC	Men:≥110cm vs.<90cm Women:≥90cm vs.<70cm	Men and women: 1.43 (1.05-1.94) Men:1.40 (0.97-2.03) Women:1.43 (0.79-2.61)	age, sex, study, alcohol, cigarette smoking, race, and BMI.
Song (2022) [33]	LC	China	Men and women (30-79years)	10.12	2529/492640	WHR	Men: ≥0.95 vs.<0.90 Women: ≥0.90 vs.<0.85	1.16 (1.04-1.29)	age at baseline, sex, residential area, education level, HBV status, diabetes, hypertension at baseline, and other lifestyle factors.
Li (2022) [36]	LC	China	Men (40-74years)	11.9	440/60625	WC	>91.8cm vs.≤77.8cm	1.65(1.04-2.60)	age, education, income, cigarette smoking, alcohol drinking, history of chronic liver diseases, history of cholelithiasis, family history of liver cancer, energy intake,physical activity and BMI.
						WHR	>0.95 vs.≤0.85	1.21 (0.85-1.71)	
Wei (2018) [37]	LC	China	Men	8.9	346/104825	WC	≥95.0cm vs.85.0-89.9cm	1.98 (1.39-2.82)	age, education leve, dust exposure, smoking, alcohol drinking, diabetes, HBsAg, BMI.
Pang (2019) [38]	LC	China	Men and women (30-79years)	10	2847/512713	WC	≥100cm vs.<70cm	2.10 (1.46-3.02)	age at baseline, education, household income,smoking status, alcohol, self-rated health, and family history of cancer,BMI.
						WHR	Q5 vs. Q1	1.28 (1.11-1.47)	
Schlesinger (2013) [39]	LC	USA	Men and women	8.6	177/359525	WC	Men:≥104.5cm vs.<85.8cm Women:≥91.6cm vs.<70.6cm	1.60 (0.92-2.80)	Age, sex, education, smoking status, alcohol consumption, height, weight change was additionally adjusted for weight at age 20 (continuous), hip circumference, and waist-to-height ratio for weight (continuous).
						WHR	Men:≥0.98vs.<0.90 Women:≥0.83 vs.<0.76	1.42 (0.89-2.27)	
Li (2021) [40]	LC	China	Women (40-70 years)	17.5	241/69296	WC	Q4 vs. Q1	1.52 (1.03-2.25)	age, education, income, menopausal status,age at menarche, history of chronic hepatitis, history of cholelithiasis, family history of liver cancer, total energy intake and total physical activity.
						WHR	Q4 vs. Q1	1.32 (0.90-1.94)	
Florio (2020) [41]	LC	USA	Men and women	ND	2208/1167244	WC	Men:≥110cm vs.<90cm Women:≥ 90cm vs.<70cm	1.88 (1.44-2.47)	Age,race,sex,alcohol consumption ,cigarette smoking,and study.
						WHR	Men:≥0.95 vs.<0.85 Women:≥0.90 vs.<0.80	1.29 (1.04-1.60)	
Steffen (2015) [42]	GC	European countries	Men and women (25-70 years)	11	417/391456	WC	Q5 vs. Q1	GCA:1.91 (1.09-3.37) GNCA:1.25 (0.75-2.08)	BMI,sex,education,smoking habits, alcohol consumption at recruitment and amount of alcohol, physical activity and intake of red and processed meat, vegetables, citrus and non-citrus.
						WHR	Q5 vs. Q1	GCA:1.95 (1.12-3.38) GNCA:2.05 (1.19-3.52)	
O’Doherty (2012) [43]	GC	USA	Men and women (50-71 years)	9	316/218854	WC	Q4 vs. Q1	GCA:1.98 (1.11-3.53) GNCA:1.46 (0.71-3.03)	Age, sex,total energy, antacid use,aspirin use, non-steroidal antiinflammatory drug use ,marital status, diabetes, cigarette smoking, education, ethnicity, alcohol consumption, physical activity, red and white meat intake, and fruit and vegetable intake.
						WHR	Q4 vs. Q1	GCA:1.08 (0.71-1.63); GNCA:1.46 (0.86-2.48)	
Lin (2015) [44]	GC	Norway	Men and women (≥20 years)	10.6	373/192903	WC	Men:≥94cmvs.<94cm; women:≥80cm vs.<80cm	GC:1.47 (1.14-1.90)	Age,sex ,BMI, education, smoking status and family cancer history.
Sanikini (2020) [45]	GC	UK	Men and women (40-69 years)	6.5	229/458713	WC	>96cm vs. <84cm	GCA:1.28 (0.70-2.32) GNCA:0.96 (0.51-1.81)	age (5 year categories), sex, Townsend deprivation index (quintiles), recruitment assessment centre , smoking status, education and alcohol intake.
						WHR	>0.92 vs <0.83	GCA:1.01 (0.49-2.09) GNCA:1.10 (0.52-2.34)	
Choi (2021) [46]	GC	Korea	Women (≥40years)	7.2	42441/6272367	WC	90cm vs.≥65-74.9cm	Premenopausal women: 1.02 (0.81–1.27) Postmenopausal women: 1.14 (1.09–1.19)	age at menarche, parity, duration of breastfeeding, duration of oral contraceptive use in premenopausal women and duration of hormone replacement therapy and age at menopause in postmenopausal women.
Steffen (2015) [42]	EC	European countries	Men and women (25-70 years)	11	124/391456	WHR	Q5 vs. Q1	EAC:4.05 (1.85-8.87)	BMI, sex, education, smoking habits, alcohol consumption at recruitment and amount of alcohol, physical activity and intake of red and processed meat, vegetables, citrus and non-citrus.
O’Doherty (2012) [43]	EC	USA	Men and women (50-71 years)	9	253/218854	WC	Q4 vs. Q1	EAC: 2.03 (1.21-3.39)	Age, sex,total energy, antacid use, aspirin use, non-steroidal antiinflammatory drug use, marital status, diabetes, cigarette smoking, education, ethnicity, alcohol consumption, physical activity, red and white meat intake,and fruit and vegetable intake.
						WHR	Q4 vs. Q1	EAC: 1.47 (0.99-2.18)	
Lin (2015) [44]	EC	Norway	Men and women (≥20 years)	10.6	126/192903	WC	Men:≥94 cmvs. <94 cm; Women:≥80cm vs. <80cm	EAC:2.48 (1.27–4.85) EACC:1.19 (0.71-2.00)	Age, sex, BMI, education,smoking status and family cancer history.
Sanikini (2020) [45]	EC	UK	Men and women (40-69 years)	6.5	466/458713	WC	>96 vs <84 cm	EAC:2.30 (1.47–3.57) EACC:0.55 (0.32–0.95)	age (5 year categories), sex, Townsend deprivation index (quintiles), recruitment assessment centre , smoking status, education and alcohol intake.
						WHR	>0.92 vs <0.83	EAC: 1.71 (1.01–2.90) EACC:1.03 (0.55-1.91)	
Wang (2008) [47]	CRC	USA	Men and women(≥45years)	7.7	953/95151	WC	Men:≥120cm vs.<95cm Women:≥110cm vs. <85cm	CRC: Men:1.68 (1.12-2.53) Women:1.75 (1.20-2.54) CC: Men:2.05 (1.29-3.25) Women:1.54 (1.00-2.37) RC: Men:1.02 (0.43-2.42) Women:2.65 (1.23-5.71)	height, education, physical activity, smoking, alcohol intake, NSAID use, multivitamin use, and history of colorectal endoscopy (women+HRT use).
Moore (ages 30-54) (2004) [48]	CRC	USA	Men and women (30-54years)	51	157/3764	WC	Men:≥101.6cm vs.<83.8cm Women: ≥99.1cm vs.<81.3 cm	CC: Men and women: 2.9 (1.2-6.7) Men:3.3 (0.91-12.3) Women:2.3 (0.74-7.0)	BMI, sex, education, age, height, alcohol intake, cigarettes per day, physical activity.
Moore (ages 55-79) (2004) [48]	CRC	USA	Men and women (55-79years)	51	149/3802	WC	Men:≥101.6cm vs.<83.8cm Women: ≥99.1cm vs.<81.3 cm	CC: Men and women: 2.4 (1.0-5.6) Men:3.0 (0.86-10.3) Women:2.1 (0.63-6.7)	BMI, education, age, height, alcohol intake, cigarettes per day, physical activity.
Maclnnis (2004) [49]	CRC	Australia	Men (27-75years)	12	153/16556	WC	Men:>99.3cm vs.<87.0cm	CC:Men 2.1 (1.3-3.5)	age at attendance, country of birth, highest level of education.
						WHR	Men:>0.96 vs.<0.88	CC: Men 2.1(1.3-3.4)	
Maclnnis (2006) [50, 51]	CRC	Australia	Wonmen (27-75years)	12	212/24072	WC	Women:≥88cm vs. <80cm	CC: Women 1.4(1.0-1.9)	country of birth, highest level of education, hormone replacement therapy use.
						WHR	Women:≥0.80 vs.<0.75	CC: Women 1.7(1.1-2.4)	
MacInnis (2006) [50, 51]	CRC	Australia	Men and women (27-75years)	13	229/4114	WC	Men: ≥102cm vs.<94cm Women: ≥88cm vs. <80cm	RC: Men and women: 1.4(1.0-1.9) Men: 1.4 (0.9-2.2) Women: 1.4 (0.8-2.2)	age as the time axis, sex, and country of birth.
						WHR	Men: ≥0.95 vs.<0.90 Women:≥0.80 vs.<0.75	RC: Men and women: 1.3 (0.9-1.8) Men:1.2 (0.8-1.8) Women:1.4 (0.8-2.4)	
Pischon (2006) [52]	CRC	Europe	Men and women (25-70years)	8	1570/368277	WC	Men:≥103.0cm vs.<86.0cm Women: ≥89.0cm vs.<70.2cm	CC: Men:1.39 (1.01-1.93) Women:1.48 (1.08-2.03) RC: Men:1.27 (0.84-1.91) Women:1.23 (0.81-1.86)	age, center and age at recruitment, smoking status, education, alcohol intake, physical activity, fiber intake, consumption of red and processed meat, fish and shellfish, fruits and vegetables, height.
						WHR	Men:≥0.990 vs.<0.887 Women:≥0.846 vs. <0.734	CC: Men:1.51 (1.06-2.15) Women:1.52 (1.12-2.05) RC: Men:1.93 (1.19-3.13) Women:1.20 (0.81-1.79)	
Oxentenko (2010) [53]	CRC	USA	Women (55-69years)	19	1464/36941	WC	Women: ≥96.53cm vs.≤77.15cm	CRC: Women 1.32(1.11-1.56)	age at baseline, age at menopause, exogenous estrogen use, oral contraceptive use, smoking status, cigarette pack-years, physical activity level, selfreported diabetes mellitus, and intake of total energy, total fat, red meat, fruits and vegetables, calcium, folate, vitamin E and alcohol.
						WHR	Women: ≥0.90 vs.≤0.78	CRC: Women 1.28(1.08-1.50)	
Li (2013) [54]	CRC	China	Men: (40-74years) Women: (40-70years)	Men:11 Women: 5.5	935/134255	WC	Men:≥92cm vs.<78cm Women: ≥85cm vs.<70cm	CRC: Men:1.38 (0.97-1.97) Women:1.26 (0.93-1.72) CC: Men:2.00 (1.21-3.29) Women:1.34 (0.89-2.00) RC: Men 0.88 (0.52-1.49) Women 1.17 (0.73-1.88)	age at baseline, education, income, pack-years of cigarette use, tea consumption, alcohol consumption, physical activity, family history of colorectal cancer and intakes of total energy, red meat, fruits and vegetables.
						WHR	Men:≥0.95 vs.<0.85 Women:≥0.85 vs.<0.77	CRC: Men:1.65 (1.12-2.41) Women:1.01 (0.79-1.31) CC: Men:1.97 (1.19-3.24) Women:0.96 (0.69-1.34) RC: Men 1.24 (0.69-2.26); Women 1.11 (0.74-1.66)	
Andreasson (2019) [55]	CRC	Swedish	Men and Women	21.5	937/27504	WC	Men:≥102cm vs.102cm Women:≥88cm vs.88cm	CRC: Men:1.45 (1.17-1.80) Women:1.01 (0.80-1.29) CC: Men:1.49 (1.13-1.96) Women:0.97 (0.72-1.31) RC: Men:1.33 (0.93–1.89) Women:1.15 (0.76-1.73)	age, alcohol, smoking, higher education and physical activity.
						WHR	Men:≥0.90 vs. 0.90 Women:≥0.85 vs.0.85	CRC: Men:1.42 (1.18-1.72) Women: 1.00 (0.78-1.30) CC: Men:1.4 (1.12-1.82) women:0.82 (0.59-1.15) RC: Men:1.36 (1.01-1.85) Women:1.39 (0.92-2.01)	
Larsson (2006) [55]	CRC	Swedish	Men: (45-79years)	7.1	496/45906	WC	Men:≥104cm vs.<88cm	CRC: Men:1.29 (0.90-1.85) CC: Men:1.44 (0.93-2.24) RC: Men:1.24 (0.68-2.25)	age, education, family history of colorectal cancer, history of diabetes, smoking, aspirin use, leisure-time physical activity, height.
Park (2012) [57]	CRC	UK	Men and women (40-79years)	11	357/24244	WC	Men:≥103.3cm vs.<88.0cm Women: ≥90.5cmvs.<73.0cm	CRC: Men:0.86 (0.55-1.36) Women:1.65 (0.97-2.86)	age, sex, smoking, alcohol, education, exercise, family history of CRC, energy intake, folate, fibre, total meat and processed meat, intakes, height.
						WHR	Men:≥0.979 vs.<0.883 Women:≥0.844 vs.<0.739	CRC: Men:1.34 (0.79-2.25) Women:2.07 (1.17-3.67)	
Martinez (1997) [58]	CRC	USA	Women (30-55years)	12	212/67802	WC	Women:>34in vs.≤27.5in	CC: Women:1.48 (0.89-2.46)	age, cigarette smoking, family history of colorectal cancer, leisure-time physical activity, postmenopausal hormone use, aspirin use, intake of red meat, and alcohol consumption.
						WHR	Women: >0.833 vs. <0.728	CC: Women:1.48 (0.88-2.49)	
Giovannucci (1995) [59]	CRC	USA	Men: (40-75years)	5	205/47723	WC	Men:≥43in vs.<35in	CC: Men:2.56 (1.33-4.96)	age, history of endoscopic screening, previous polyp diagnosis, parental history of colorectal cancer, pack-years of smoking, physical activity, aspirin use, and intake of folate, methione, alcohol, dietary fiber, total energy, and red meat.
						WHR	Men:≥0.99 vs. <0.90	CC: Men:3.41 (1.52-7.66)	
Kabat (2015) [60]	CRC	USA	Women (50-79years)	12.7	1908/143901	WC	Q5 vs. Q1	CRC: Women:1.90 (1.61–2.25)	age , alcohol, smoking, hormone therapy, MET hours/week, aspirin intake, diabetes, family history of colorectal cancer in a first degree relative, education, ethnicity, treatment allocation.
						WHR	Q5 vs. Q1	CRC: Women:1.65 (1.40–1.93)	
Folsom (2000) [61]	CRC	USA	Women (55-69years)	11	462/31702	WC	Women: ≥96.0cm vs. <74.3cm	CC: Women:1.6 (1.2-2.2)	age, educational level , physical activity, alcohol intake, smoking status, pack-years of cigarette smoking(continuous),age of first live birth, estrogen use, vitamin use, and energy, whole grain, fruit and vegetable, fish, and red meat intake and Keys score.
						WHR	Women:≥0.901 vs.<0.762	CC: Women:1.2 (0.9-1.7)	
Keimling (2013) [62]	CRC	USA	Men and women(50-71years)	10	2869/203177	WC	Men: ≥106.5cm vs.<89.5cm Women: ≥94.5cm vs. <73.6cm	CC: Men:1.45 (1.16-1.82) Women;0.90 (0.63-1.27) RC: Men:0.97 (0.67-1.38) Women:1.0 1(0.53-1.94)	age, education, race/ethnicity, smoking status, marital status, physical activity, NSAID use, family history of colorectal cancer, diabetes status, dietary intakes of total energy, fiber, folate, calcium, red meat, fruits and vegetables, alcohol, HRT, height(WC+ hip circumference).
						WHR	Men: ≥ 1.000 vs.<0.898 Women: ≥ 0.877 vs. <0.746	CC: Men:1.29 (1.10-1.52) Women0.90 (0.70-1.15) RC: Men:1.08 (0.82-1.43) Women:1.13 (0.69-1.86)	
Schoen (1999) [63]	CRC	USA	Men and women (≥65years)	6.4	102/5849	WC	Men:104.1-145.5cm vs.69-91cm Women:101.2-167cm vs.32.5-82cm	CRC: 2.2 (1.2-4.1)	age, sex, and physical activity.
						WHR	Men: 0.61-0.93 vs. 1.01-2.33 Women: 0.961-2.06 vs.0.61-0.83	CRC: 2.6 (1.4-4.8)	
Ahmed (2006) [64]	CRC	USA	Men and women(45-64years)	11.5	194/14109	WC	Men:≥102cm vs.<102cm Women:≥88cm vs.<88cm	CRC: 1.40 (1.0-1.9)	family history of colorectal cancer, physical activity, nonsteroidal antiinflammatory, drug use, aspirin use, pack years of cigarette use, and grams of alcohol per week(women+HRT use).
Ortega (2017) [65]	CRC	UK	Men and women(40-69years)	5.6	2636/472526	WC	Men:≥105cm vs.<88cm Women:≥95cm vs.<74cm	CRC: Men:1.66 (1.39–1.99) Women:1.22 (0.99-1.52) CC: Men:1.89 (1.49–2.40) Women:1.26 (0.97-1.62) RC: Men: 1.40 (1.06–1.86) Women 1.20 (0.79–1.81)	physical activity, smoking status and intensity, alcohol consumption frequency, family history of colorectal cancer, prevalent diabetes, and stratifed by age (5-year categories), Townsend deprivation index ffhs, and region of the recruitment assessment centre.
						WHR	Men:≥0.99vs. <0.88 Women:≥0.88 vs.<0.76	CRC: Men:1.70 (1.43–2.02) Women:1.33 (1.08-1.65) CC: Men:1.73 (1.35–2.21) Women:1.29 (1.01-1.65) RC: Men: 1.42 (1.05–1.91) Women : 1.50 (0.98–2.28)	
Lu (2016) [66]	CRC	Norway	Men and women	16	2044/143477	WC	Men: ≥96cm vs.<88cm Women: ≥86 vs. <75cm	CRC: Men and women: 1.56 (1.32-1.84) Men:1.4 (1.1-1.7) Women:1.81 (1.39-2.36)	education, smoking status, alcohol drinking, physical activity, family history of cancer, study center, and/or anthropometrics when appropriate, stratified by age groups.
Tran (2022) [67]	CRC	Korea	Men and women	9.4	128/34800	WC	Men:≥90cm vs.<90cm Women:≥85cm vs.<85cm	CRC: Men and women: 1.18 (0.83-1.68) Men:1.29 (0.82-2.01) Women:1.01(0.55-1.85)	sex,age, alcohol consumption, smoking status,regular exercise, monthly income, marital status,EEand a first-degree family history of CRC.
Wong (2019) [68]	CRC	Asia	Women	3	616/28191	WC	Women: > 87.2 vs. < 74.1cm	CRC: 1.62 (1.17 - 2.25) CC: 2.14 (1.42 –3.25) RC: 1.02 (0.59-1.76):	Age, housing type, race, Body Mass Index.
						WHR	Women: > 0.87 vs. < 0.77	CRC: 1.44 (1.14-1.83) CC: 1.74 (1.30 -2.34) RC: 1.03 (0.68 -1.56)	
Song (2016) [69]	CRC	USA	Men and women	23-24	1884/112610	WC	Q5 vs. Q1	CRC: Men:0.85 (0.66–1.11) Women:1.64 (1.17–2.29)	Age, height, family history of colorectal cancer, pack-years of smoking, multivitamin use, physical activity, alcohol consumption, calcium intake, AHEI score, Body Mass Index.
						WHR	Q5 vs. Q1	CRC: Men:1.05 (0.86–1.29) Women: 1.33 (1.03–1.71)	

*LC* liver cancer, *PC* pancreatic cancer, *CRC* colorectal cancer, *RC* rectum cancer, *CC* colon cancer, *GC* gastric cancer, *GCA* gastric cardia adenocarcinoma, *GNCA* gastric non-cardia adenocarcinoma, *EC* esophageal cancer, *EAC* esophageal adenocarcinoma, *ESCC* esophageal squamous-cell carcinoma, *BMI* body mass index, *WC* waist circumference, *WHR* waist-to-hip ratio, relative risk, *95% CI* RR, 95% confidence interval

**Table 2:** Quality assessment according to the nine-star Newcastle-Ottawa Scale (NOS)

Study	Selection				Comparability		Outcome			Total stars
	1	2	3	4	5	6	7	8	9	
Stolzenberg (2008) [27]	*	*	-	*	*	*	-	*	*	7
Berrington (2006) [28]	*	*	-	*	*	*	*	*	*	8
Luo (2008) [29]	-	*	*	*	*	*	*	*	*	8
Larsson (2005) [30]	*	*	-	*	*	*	*	*	*	8
Arthur (2018) [31]	-	*	*	*	*	*	*	*	*	8
Andersson (2016) [32]	*	*	-	*	*	*	*	*	-	7
Song (2022) [33]	*	*	-	*	*	*	*	*	-	7
Campbell (2016) [35]	*	*	-	*	*	*	*	*	*	8
Li (2022) [36]	*	*	*	*	*	*	*	*	*	9
Wei (2018) [37]	-	*	*	*	*	*	-	*	*	7
Pang (2019) [38]	*	*	*	*	*	*	*	*	*	9
Schlesinger (2013) [39]	*	*	*	*	*	*	*	*	*	9
Li (2021) [40]	*	*	*	*	*	*	*	*	*	9
Florio (2020) [41]	*	*	-	*	*	*	-	*	-	8
Steffen (2015) [42]	*	*	*	*	*	*	*	*	*	9
O’Doherty (2012) [43]	-	*	-	*	*	*	*	*	*	7
Lin (2015) [44]	*	*	*	*	*	*	*	*	*	9
Sanikini (2020) [45]	-	*	*	*	*	*	*	*	*	8
Choi (2021) [46]	-	*	-	*	*	*	*	*	*	7
Hwang (2021) [34]	-	*	-	*	*	*	*	*	*	7
Wang (2008) [47]	*	*	-	*	*	*	*	*	*	8
Moore (2004) [48]	*	*	*	*	*	*	*	*	-	8
Maclnnis (2004) [49]	*	*	*	*	*	*	-	*	*	8
Maclnnis (2006) [50, 51]	*	*	*	*	*	*	-	*	*	8
Maclnnis (2006) [50, 51]	*	*	*	*	*	*	-	*	*	8
Pischon (2006) [52]	*	*	*	*	*	*	-	*	*	8
Oxentenko (2010) [53]	*	*	-	*	*	*	*	*	*	8
Li (2013) [54]	*	*	*	*	*	*	*	*	*	9
Andreasson (2019) [55]	*	*	*	*	*	*	*	*	-	8
Larsson (2006) [56]	*	*	-	*	*	*	*	*	*	8
Park (2012) [57]	*	*	-	*	*	*	*	*	-	8
Martinez (1997) [58]	-	*	-	*	*	*	*	*	*	7
Giovannucci (1995) [59]	*	*	-	*	*	*	-	*	*	7
Kabat (2015) [60]	-	*	*	*	*	*	*	*	*	8
Folsom (2000) [61]	*	*	*	*	*	*	-	*	*	8
Keimling (2013) [62]	*	*	-	*	*	*	*	*	*	8
Schoen (1999) [63]	-	*	-	*	*	*	*	*	*	7
Ahmed (2006) [64]	*	*	*	*	*	*	-	*	*	8
Ortega (2017) [65]	*	*	*	*	*	*	*	*	*	9
Lu (2016) [66]	*	*	-	*	*	*	-	*	*	7
Tran (2022) [67]	*	*	*	*	*	*	-	*	*	8
Wong (2019) [68]	*	*	-	*	*	*	*	*	*	8
Song (2016) [69]	-	*	-	*	*	*	*	*	*	7

### WC and DSC

**Fig. 2:**
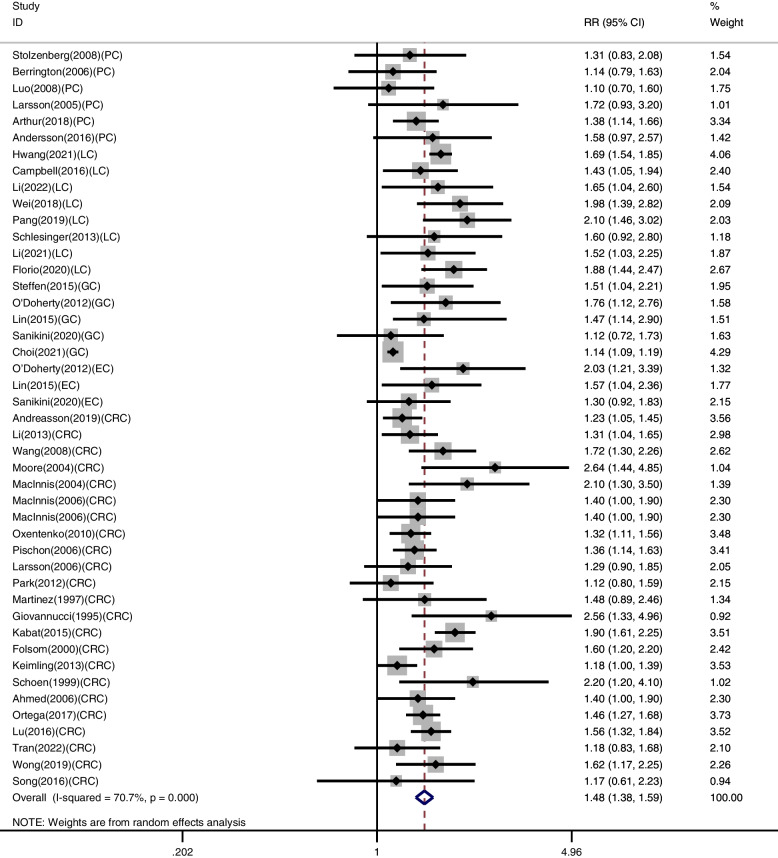
Meta-analysis of WC and risk of DSC

We included 42 studies in our meta-analysis for the association between WC and DSC risk. The forest plot is presented in Fig. 2, a higher WC can increase the incidence of DSC by 48% (RR 1.48, 95% CI 1.38–1.59, *p* < 0.001). A random-effects model was applied due to the remaining heterogeneity among the studies (I^2^ = 70.7%, *p* < 0.001). Subgroup analysis better identified the relationship between WC and the risk of DSC (Figures S1-S3). When stratified by region, associations were presented in all subgroups: North America, RR 1.54, 95% CI 1.39–1.71, *p* = 0.009; Europe, RR 1.38, 95% CI 1.29–1.48, *p* = 0.713; Asia, RR 1.52, 95% CI 1.26–1.84, *p* < 0.001; Oceania, RR 1.51, 95% CI 1.22–1.87, *p =* 0.345. When stratified by cancer type, the risk increased in all subgroups: PC, RR 1.33, 95% CI 1.16–1.53, *p* = 0.730; LC, RR 1.71, 95% CI 1.58–1.84, *p* = 0.762; GC, RR 1.29, 95% CI 1.08–1.54, *p* = 0.155; EC, RR 1.52, 95% CI 1.20–1.92, *p* = 0.362; CRC, RR 1.45, 95% CI 1.34–1.56, *p* = 0.010. There was no marked heterogeneity among subgroups, and cancer type may be the source of heterogeneity. In stratified analyses for publication year, there were all significant risk associations: before the 2010 year, RR 1.45, 95% CI 1.33–1.58, *p* = 0.299; after the 2010 year, RR 1.47, 95% CI 1.35–1.62, *p* < 0.001.

**Fig. 3:**
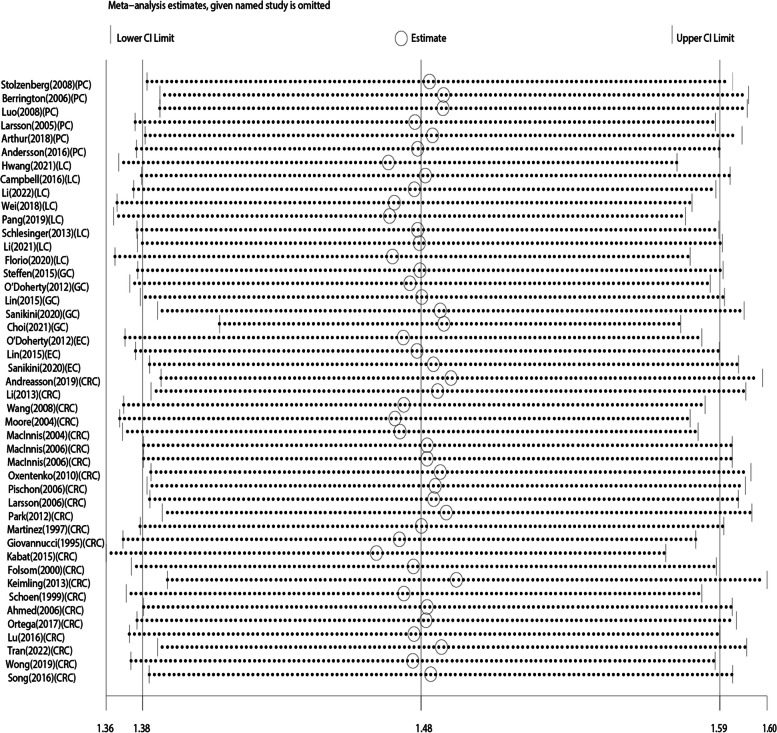
Sensitivity analysis of the association between WC and DSC

We further performed a sensitivity analysis to assess the reliability of the included articles by excluding each article individually (Fig. 3). When we excluded any studies, we did not find a clear difference, implying that the included studies were stable. Publication bias was shown in Egger’s test (*p* < 0.05), but was not evident in Begg’s test (*p* = 0.368). Therefore, we evaluated the stability of the results by trim-and-fill method, as shown in Figure S4. The trim-and-fill method resulted in an adjusted effect size of 1.24 (95% CI 1.15–1.34) after filling 21 studies. It showed that publication bias had little influence and the results were robust.

### WHR and DSC

**Fig. 4:**
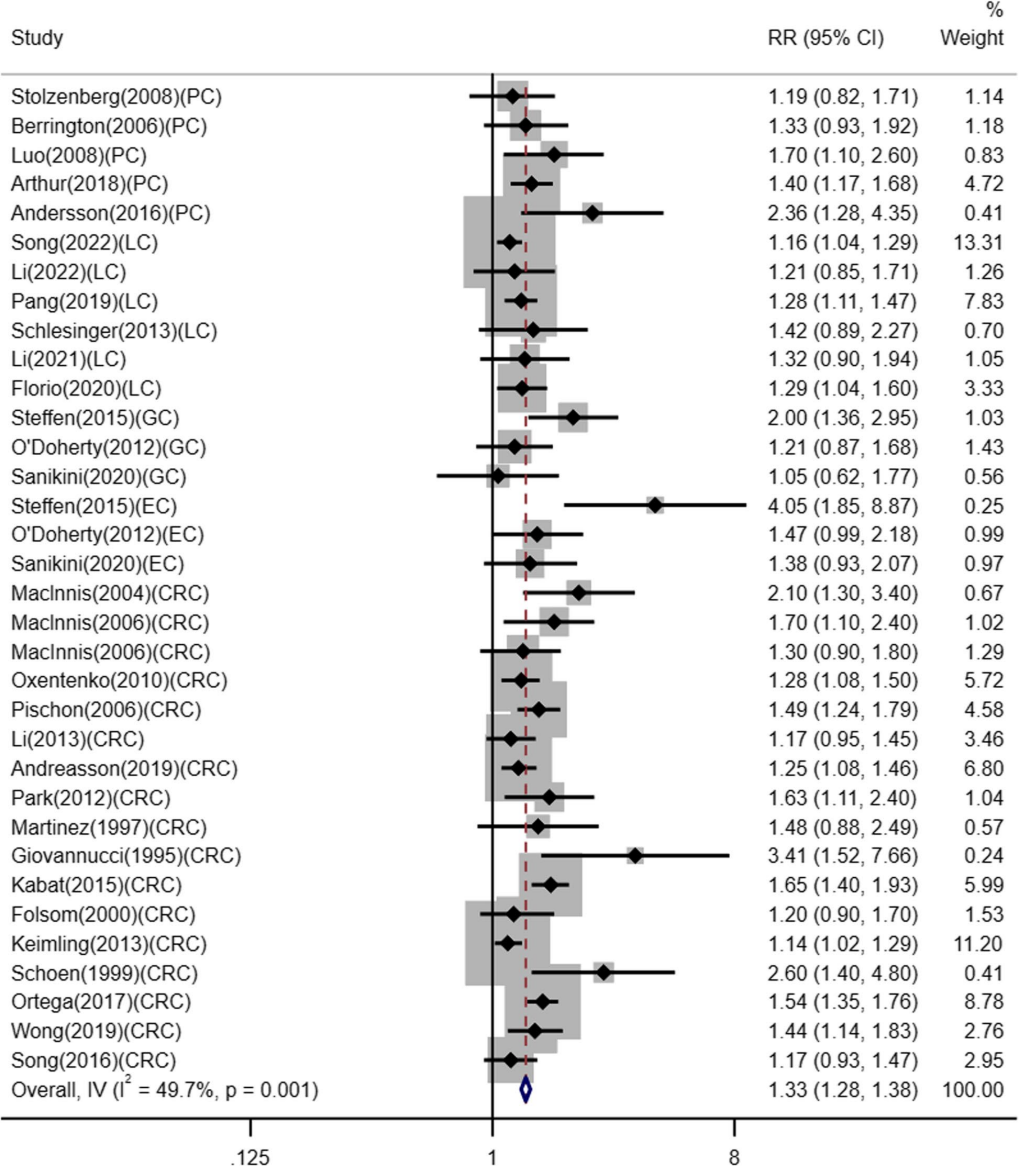
Meta-analysis of WHR and risk of DSC

**Fig. 5:**
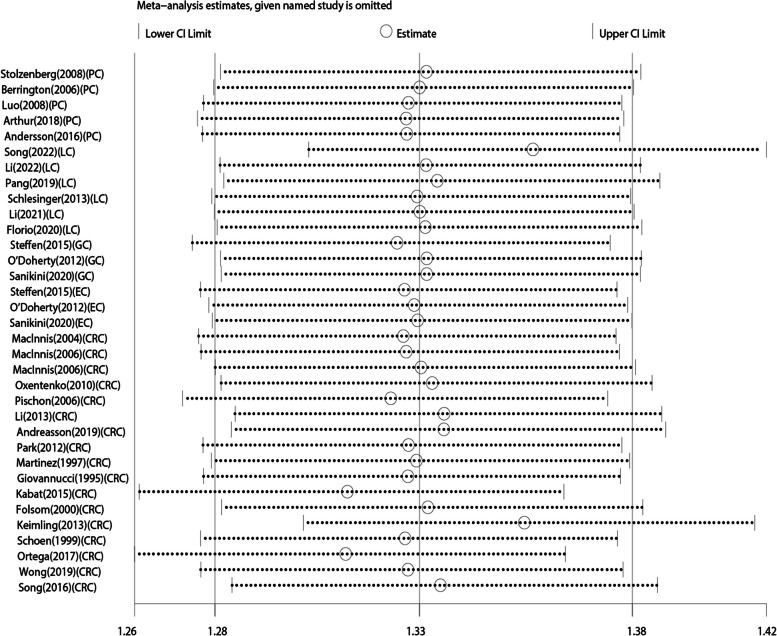
Sensitivity analysis of the association between WHR and DSC

We included 31 studies in our meta-analysis for the association between WHR and DSC risk. The forest plot is presented in Fig. 4, a higher WHR can increase the incidence of DSC by 33% (RR 1.33, 95% CI 1.28–1.38, *p =* 0.001). The fixed effects model was used because there was no significant heterogeneity between studies (*I*^2^ = 49.7%, *p =* 0.001). Subgroup analyses better defined the relationship between WHR and the risk of different cancer types of the digestive system. When stratified by cancer type, the risk increased in all subgroups: PC, RR 1.42, 95% CI 1.24–1.63, *p* = 0.360; LC, RR 1.22, 95% CI 1.13–1.32, *p* = 0.833; GC, RR 1.40, 95% CI 1.12–1.75, *p* = 0.075; EC, RR 1.60, 95% CI 1.23–2.09, *p* = 0.047; CRC, RR 1.36, 95% CI 1.29–1.43, *p* = 0.001 (Figure S5). We further performed a sensitivity analysis to assess the reliability of the included articles by excluding each article individually (Fig. 5). When we excluded any studies, we did not find a clear difference, implying that the included studies were stable. Publication bias was shown in Egger’s test (*p* < 0.05), but was not evident in Begg’s test (*p* = 3.23). Therefore, we evaluated the stability of the results by trim-and-fill method, as shown in Figure S6. The trim-and-fill method resulted in an adjusted effect size of 1.31 (95% CI 1.22–1.41) after filling 8 studies. It showed that publication bias had little influence and the results were robust.

## Discussion

In this study, we evaluated whether abdominal obesity can increase the hazard of cancer in the digestive system by analyzing existing prospective cohort studies. The study contained data from 43 cohort studies of DSC. Results showed that participants in the highest category of WC and WHR have a marked impact on developing DSC than those in the lowest category.

Results of the meta-analysis indicated that higher WHR increased the incidence of DSC by 33%, and higher WC increased the incidence of DSC by 48%. There was a greater association between WC and the risk of DSC compared with WHR. Subgroup analysis by region found that higher WC would increase the rate of digestive system cancer in North America by 54%, in Europe by 38%, in Asia by 52%, and in Oceania by 51%. Higher WC will increase the incidence of DSC in all regions. Subgroup analysis by publication time showed that higher WC is consistently related to the incidence of DSC. Subgroup analysis by cancer type showed that higher WC and WHR would increase the prevalence of LC, PC, GC, EC, and CRC. Subgroup analysis showed that WC and WHR were closely associated with the risk of different types of digestive system cancer. Consistent with the results of previous meta-analyses, abdominal obesity increased the risk of PC, LC, GC, EC and CRC [15–18].

Numerous epidemiological surveys and studies have suggested that obesity raises the danger of many diseases, including DSC [70]. A cohort study based on population showed that BMI is connected with 17 malignancies, including liver, colon, esophagus, stomach, and other digestive systems [71]. Most researchers are committed to studying the effect of common obesity in some malignant tumors, and less attention has been paid to the potential harms of abdominal obesity. The mechanism of obesity-promoting cancer in different digestive organs is different [72–78]. The mechanism of obesity in CRC may be that the first obesity provoked the microecological imbalance of the gut, resulting in the increase of the permeability of the intestinal epithelial cells to the microbial product. So the immune cells that live here secrete inflammatory factors that stimulate the growth of colorectal cancer cells. On the other hand, obesity has been able to stimulate the intestinal mucosa, allowing food metabolites to enter the gut. It causes insulin resistance and high blood sugar in the body, which promotes the occurrence of tumors [72–74]. One possible mechanism of obesity in pancreatic cancer is that autophagy can cause endoplasmic reticulum stress and damage to pancreatic cells after cell death, eventually leading to inflammation. With metabolic changes and increased autophagy, the progression of pancreatic intraepithelial tumors and pancreatic ductal adenocarcinoma can promote cell proliferation [76, 77]. Obesity can also promote LC along with hepatitis virus infection, leptin, and other risk factors [78].

BMI is the traditional indicator of assessing obesity in clinical, but it is controversial about the correlation between obesity and health risks. Abdominal obesity is the main independent dangerous factor for developing heart metabolic diseases [79]. WC and WHR represent exactly the parameters of abdominal obesity, so they can better predict the health risks associated with obesity. In predicting high blood pressure and metabolic syndrome, an observational study found that WC was significantly better than BMI [80]. The trait has been demonstrated in postmenopausal women [81]. In addition, available epidemiological evidence showed WC and WHR may predict the risk of cancer than the overall obesity of the BMI [37, 39, 48, 49]. The results of the systematic review of obesity and cancer risk by De et al. also suggested that abdominal obesity was more likely to predict the risk of GC and CRC than BMI [82]. The reason for this may be that BMI does not assess the distribution of fat mass, as well as not distinguishing whether it is fat mass or muscle mass that causes obesity [17]. Visceral fat can produce systemic endocrine effects due to its metabolic activity [83]. WC and WHR are considered to be better predictors of cancer development risk. Therefore, although some meta-analyses proved a positive connection between digestive system cancers and BMI, our meta-analysis can better explore digestive cancers by using WC and WHR.

### Strengths and limitations

In our meta-analysis, the expanded sample size, and enriched diversity of ethnic and geographic backgrounds may improve the ability to find important associations and afford more accurate estimations of effect. Prospective cohort researches were contained in this study, so our study was based on high strength of etiological evidence with an important theoretical basis. And it can effectively avoid selection bias and recall bias. Almost all studies have regulated significant covariants, containing age, gender, educational level, alcohol consumption, smoking, and so on. With the intention of reducing the confounding bias as much as possible. And all the researches contained in the meta-analysis were of high quality assessed by NOS. Therefore, the conclusion of our study has high reliability.

However, several limitations should also be considered in our meta-analysis. Firstly, the studies included in this meta-analysis were from different regions and races, which may have a high impact on the findings. And although estimates were adjusted across studies, different variables were used in different studies for adjusted estimates, which may be a source of heterogeneity. Moreover, due to the observational design of the included studies, unmeasured or uncontrolled confounding factors in the original studies that affected the results may bias the pooled estimates. Secondly, most of the research did not provide a risk estimate for the changes in the WC and WHR changes, it is difficult to exclude the impact of WC and WHR changes on the results during follow-up. Thirdly, each study defines different boundaries between high and low WC and WHR categories, which may lead to greater heterogeneity in the results. The fourth limitation is although we clearly show an important effect of abdominal obesity on DSC by comparing WC or WHR for the highest and lowest categories. However, comparing WC or WHR in the highest category with the lowest category may overestimate the effect of abdominal obesity on DSC. Later we may consider a correlation dose meta-analysis to precisely characterize the relationship between abdominal obesity and DSC. Fifthly, some of the original studies did not distinguish between genders and age groups, and we could not perform subgroup analyses according to the gender and age groups of the sample to clarify whether gender age would affect the overall positive relationship between abdominal obesity and DSC. Further studies need to focus on the impact of gender differences on this association. Sixthly, WC and WHR were self-reported and self-measured in some studies, which may be subject to error and affect the association between abdominal obesity and DSC. Finally, although there was no evidence of publication bias in this study, and publication bias remains a concern, we cannot rule out such a bias due to the limited number of studies.

## Conclusion

This meta-analysis suggests that there is a significant positive correlation between WC, WHR and the prevalence of digestive cancers. Subgroup analysis showed that both WC and WHR were positively associated with the incidence of different types of cancers of the digestive system. It is necessary to take appropriate measures to reduce abdominal obesity. WC and WHR may be better predictors of digestive system cancers. However, the association between WC and DSC is greater, so more attention should be paid to measuring abdominal obesity with WC. This study also has limitations and biases. Therefore, more large-scale and high-quality prospective studies are needed to explore the association between abdominal obesity and DSC.

### Supplementary Information

**Additional file 1: Figure S1.** Subgroup analyses of WC and risk of DSC were performed by geographic region. **Figure S2.** Subgroup analyses of WC and risk of DSC were performed by cancer type. **Figure S3.** Subgroup analyses of WC and risk of DSC were performed by year of publication. **Figure S4.** The trim and fill graph of the association between WC and DSC. **Figure S5.** Subgroup analyses of WHR and risk of DSC were performed by cancer type. **Figure S6.** The trim and fill graph of the association between WHR and DSC.

## Data Availability

All data related to the present study are available in the manuscript.

## Abbreviations

DSC: Digestive system cancer
CRC: Colorectal Cancer
GC: Gastric cancer
LC: Liver cancer
EC: Esophageal cancer
BMI: Body mass index
WC: Waist circumference
WHR: Waist-to-hip ratio
RR: Relative risk
HR: Hazard ratio
CI: Confidence interval
NOS: Newcastle Ottawa Scale
