# Supplementary material for: Abdominal obesity and digestive system cancer: a systematic review and meta-analysis of prospective studies
Source: BMC Public Health. 2023 Nov 27;23:2343. doi: 10.1186/s12889-023-17275-2 (PMC10680266; doi:10.1186/s12889-023-17275-2)
Supplement: Supplementary file 1 — Additional file 1: Figure S1. Subgroup analyses of WC and risk of DSC were performed by geographic region. Figure S2. Subgroup analyses of WC and risk of DSC were performed by cancer type. Figure S3. Subgroup analyses of WC and risk of DSC were performed by year of publication. Figure S4. The trim and fill graph of the association between WC and DSC. Figure S5. Subgroup analyses of WHR and risk of DSC were performed by cancer type. Figure S6. The trim and fill graph of the association between WHR and DSC. [file 12889_2023_17275_MOESM1_ESM.docx]

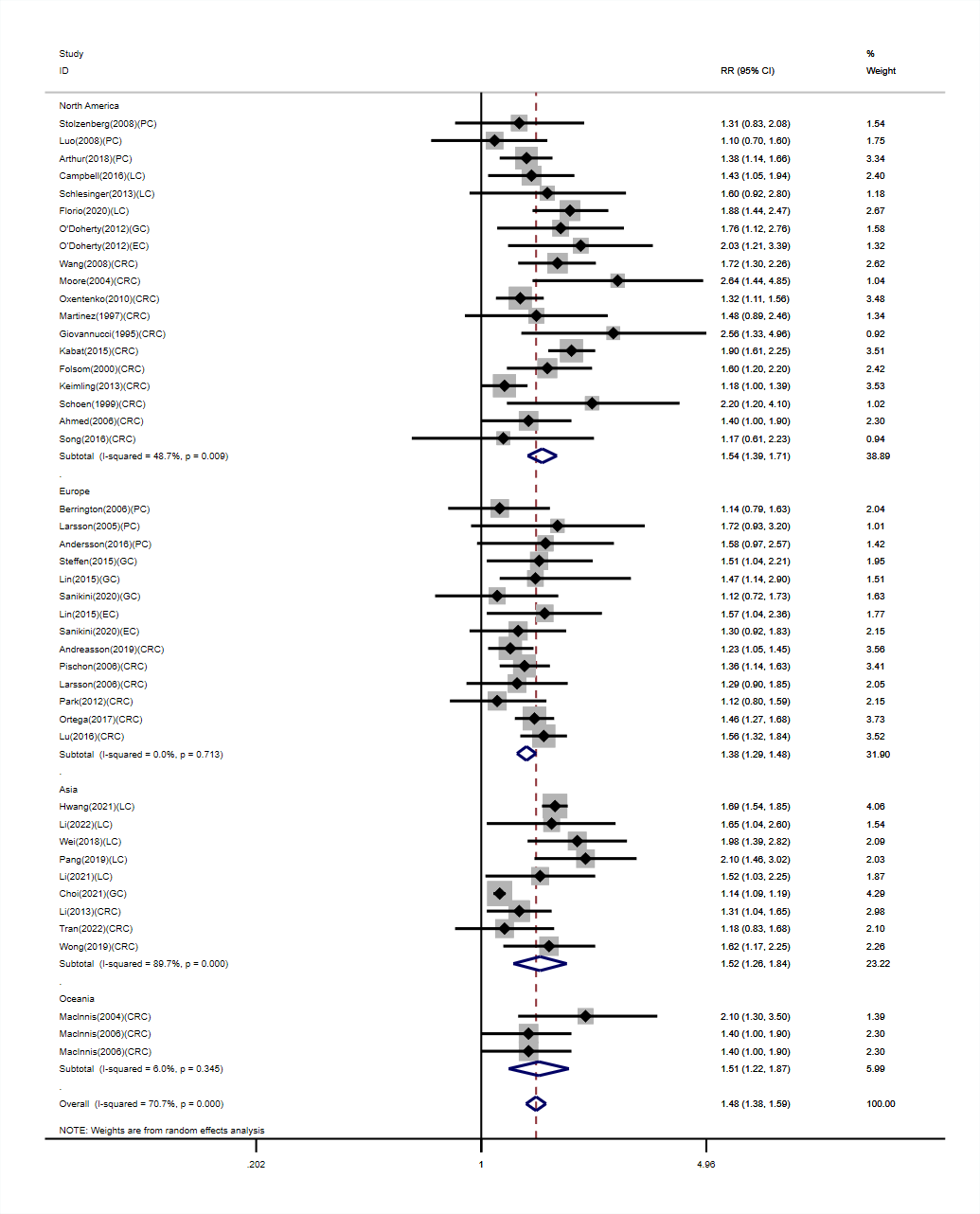
Figure S1. Subgroup analyses of WC and risk of DSC were performed by geographic region.


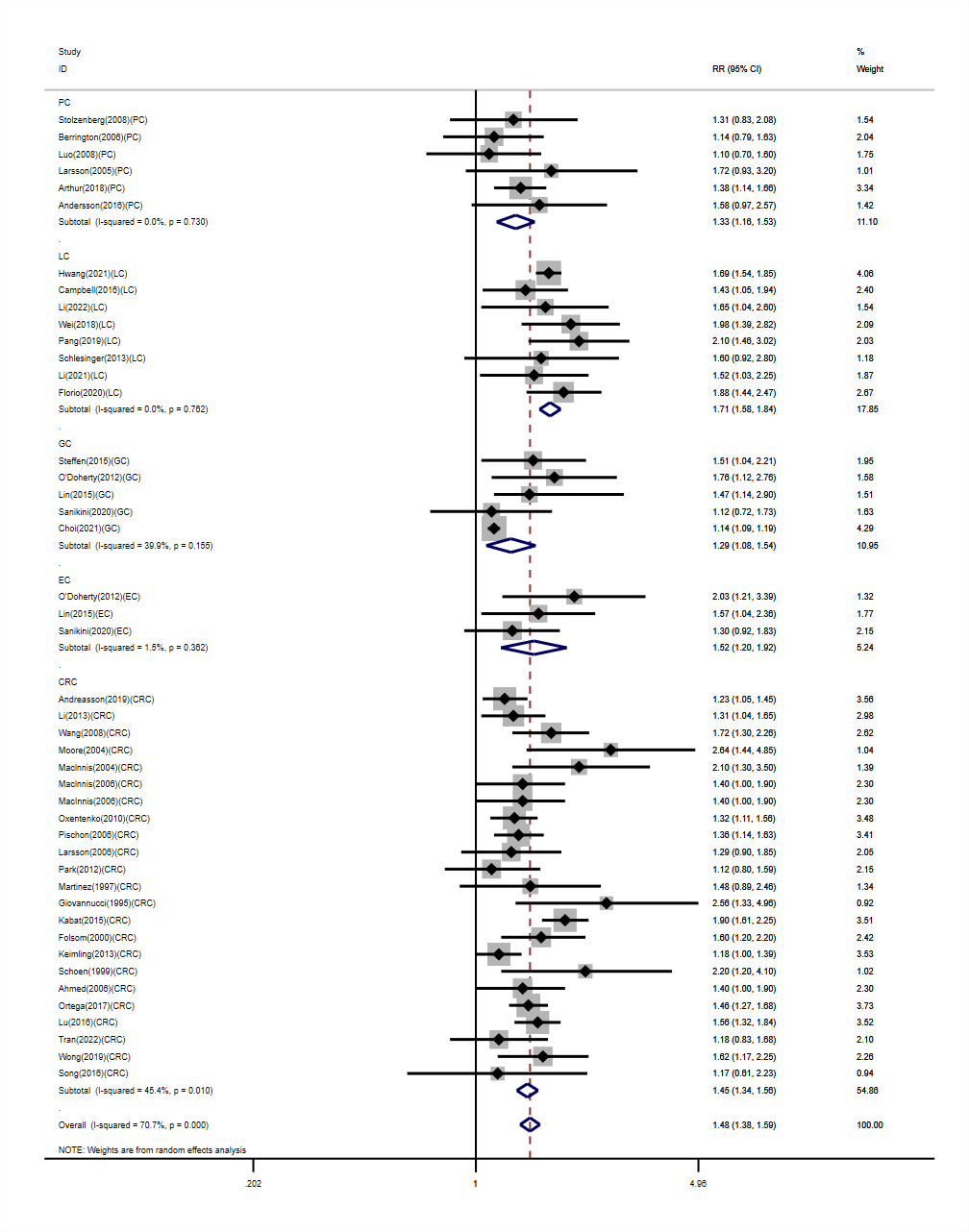


Figure S2. Subgroup analyses of WC and risk of DSC were performed by cancer type.


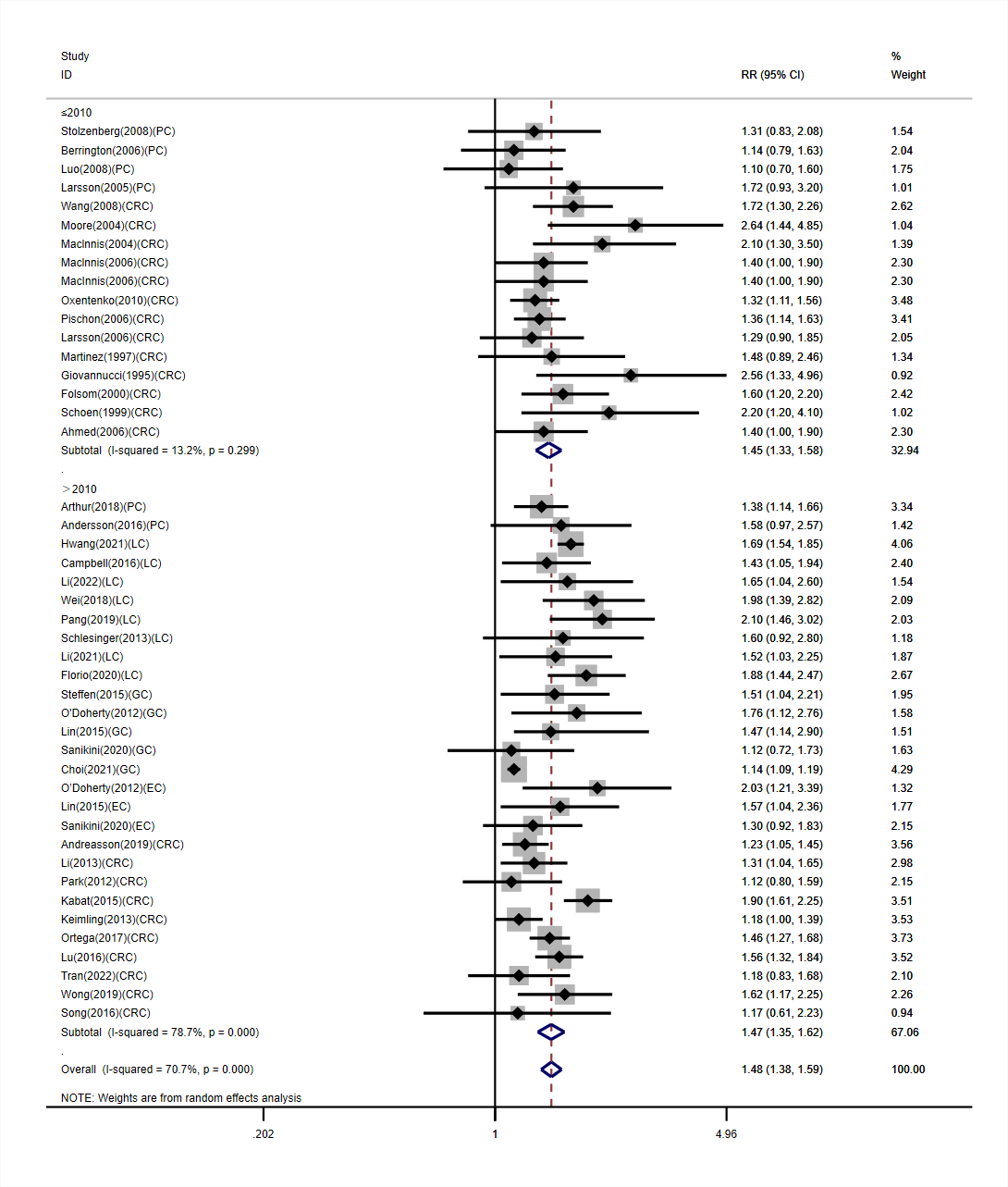


Figure S3. Subgroup analyses of WC and risk of DSC were performed by year of publication.


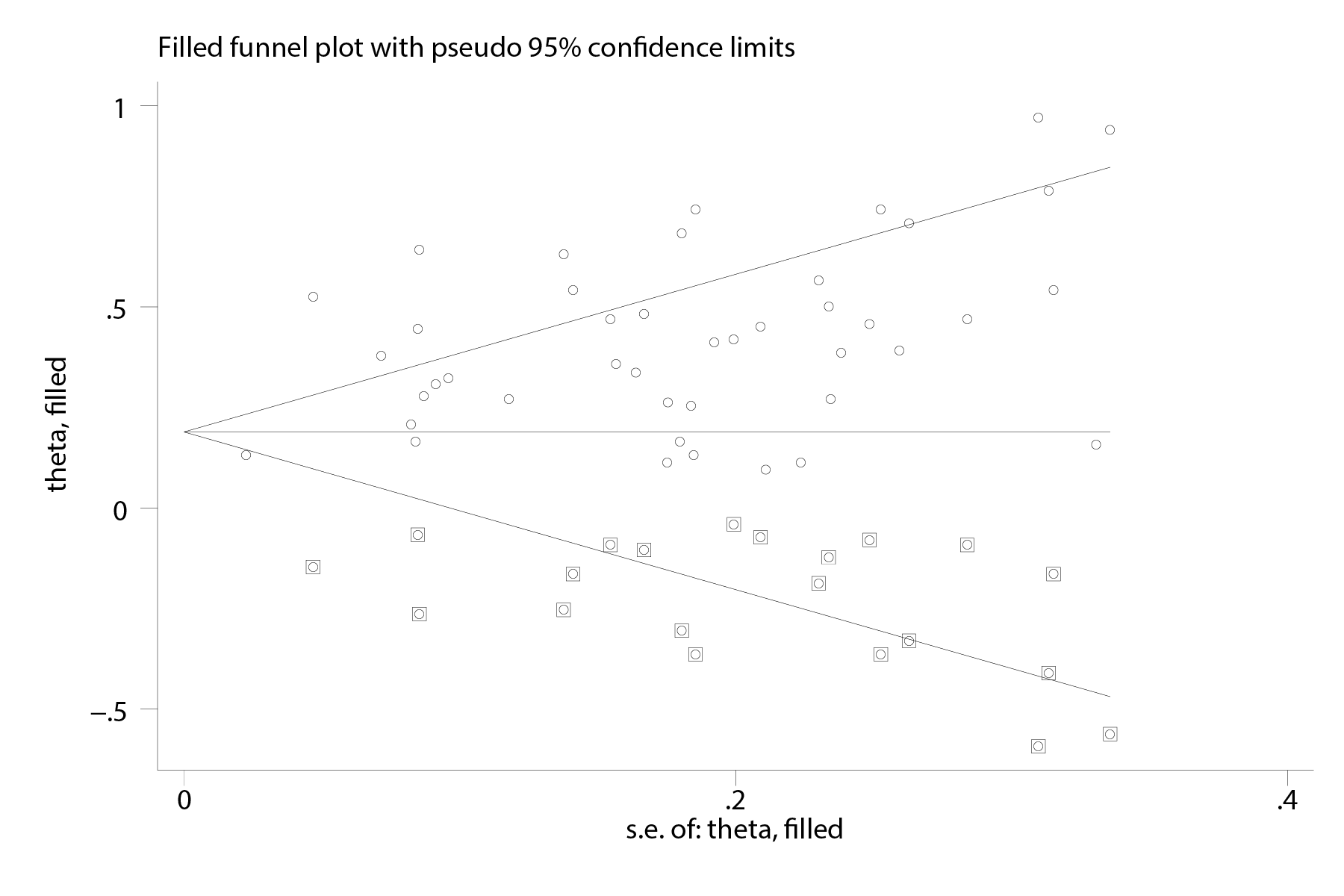


Figure S4. The trim and fill graph of the association between WC and DSC.


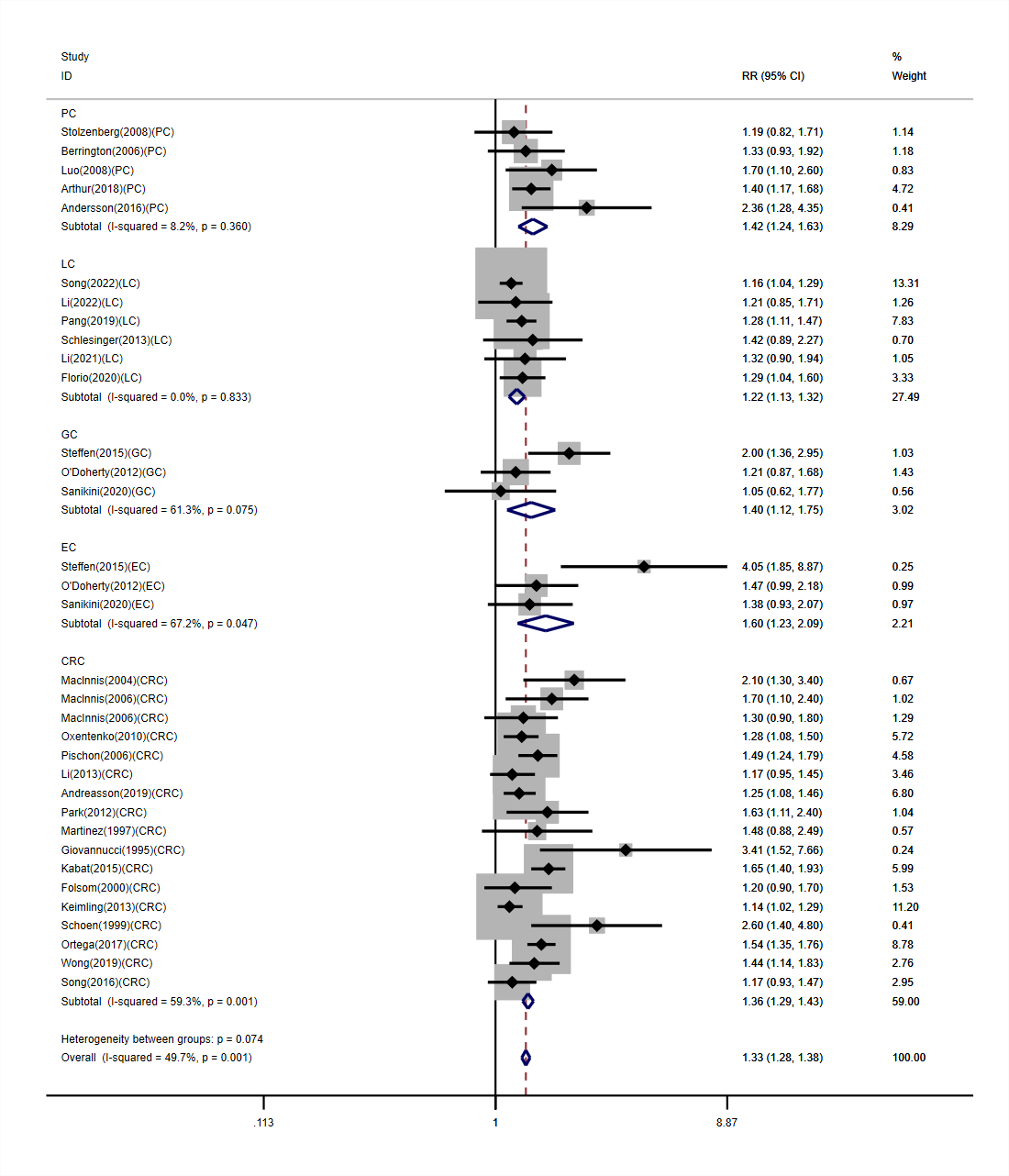


Figure S5. Subgroup analyses of WHR and risk of DSC were performed by cancer type.


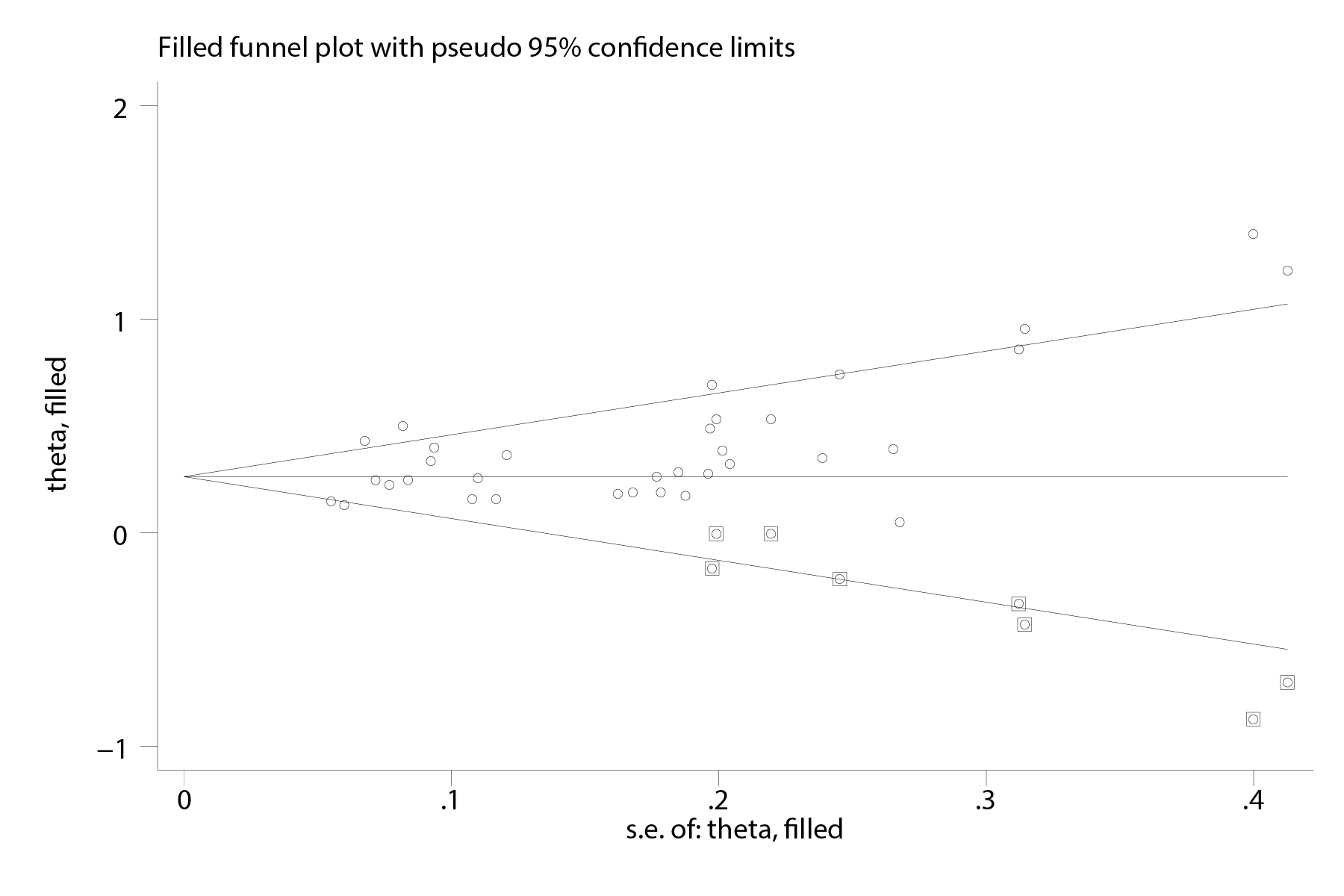
Figure S6. The trim and fill graph of the association between WHR and DSC.
